# Relationship between comprehensive geriatric assessment and amyloid PET in older persons with MCI

**DOI:** 10.1186/s12877-020-01746-x

**Published:** 2020-09-09

**Authors:** Fulvio Lauretani, Livia Ruffini, Maura Scarlattei, Marcello Maggio

**Affiliations:** 1 Department of Medicine and Surgery, University of Parma, Via Gramsci 14, 43100 Parma, Italy; 2 Cognitive and Motoric Center, Medicine and Geriatric-Rehabilitation Department of Parma, University-Hospital of Parma, 43126 Parma, Italy; 3 Nuclear Medicine Unit, University Hospital of Parma, Parma, Italy

**Keywords:** Amyloid deposition, MCI, Alzheimer’s disease, Positron emission tomography; motoric-cognitive risk syndrome, Older persons, Physical performance

## Abstract

**Background:**

The association between amyloid deposition and cognitive, behavioral and physical performance in mild cognitive impairment (MCI) due to Alzheimer’s disease (AD) has been poorly investigated, especially in older persons.

**Methods:**

We studied the in vivo correlation between the amyloid deposition at Positron Emission Tomography (amyloid-PET) and the presence of memory loss, reduced executive function, neuropsychiatric symptoms and physical performance in older persons with MCI. Amyloid-PET was performed with 18F-flutemetamol and quantitatively analyzed.

**Results:**

We evaluated 48 subjects, 21 men and 27 women. We performed in each patient a comprehensive geriatric assessment (CGA) including Mini Mental State Examination (MMSE), Clock Drawing Test (CDT), Activity Daily Living (ADL), Instrumental Activity of Daily Living (IADL), Neuropsychiatric inventory (NPI) questionnaire, 15 Geriatric Depression Scale (GDS), Short Physical Performance Battery (SPPB) and Hand Grip strength. Then, each patient underwent amyloid-PET. Mean age of the enrolled subjects was 74.6 ± 7.8 years. All of these subjects showed preserved cognitive function at MMSE > 24, while 29 of 48 subjects (61.0%) had altered CDT.

Mean NPI score was 6.9 ± 5.9. The mean value of SPPB score was 9.0 ± 2.6, while the average muscle strength of the upper extremities measured by hand grip was 25.6 ± 7.7 Kg. CT/MRI images showed cortical atrophic changes in 26 of the 48 examined subjects (54.0%), while cerebrovascular modifications were present in 31 subjects (64.5%). Pathological burden of amyloid deposits was detected in 25 of 48 (52.0%) patients with a mean value of global z-score of 2.8 (subjects defined as MCI due to AD).

After stratifying subjects in subclasses of clinical alterations, more probability of pathological amyloid deposition was found in subjects with impaired CDT and higher NPI score (O.R. = 3.45 [1.01–11.2], *p* = 0.04), with both impaired CDT and low physical performance (O.R. = 5.80 [1.04–32.2], *p* = 0.04), with altered CDT and high NPI score (O.R. = 7.98 [1.38–46.0], *p* = 0.02), and finally in those subjects with altered CDT, high NPI and low physical performance (O.R. = 5.80 [1.05–32.2], *p* = 0.04).

**Conclusion:**

Our findings support the recent hypothesis that amyloid deposition could be associated with multiple cerebral dysfunction, mainly affecting executive, behavioral and motor abilities.

## Background

Dementia in industrialized countries affects about 8% of over sixty and rises in up to 20% after the eighties [1]. According to some perspectives, dementia may triple in the next 30 years in Western countries. Therefore, the World Health Organization (WHO) considers it a “worldwide public health priority”. Globally, dementia affected about 46 million people in 2015 [2] and the number is estimated to double in 2030. In view of the high social and economic burden of the disease, all countries should include dementia in their public health programs, and develop coordinated strategies to reduce its clinical impact and related complications [3].

Alzheimer’s disease (AD) is the most common type of dementia in which early clinical manifestations are characterized by a number of cognitive and behavior symptoms [4, 5]. With the progression of the disease more cognitive and behavior changes are observed, causing a progressive loss of autonomy and, in advanced stages, also compromising motor functions with the appearance of hypokinetic-hypertonic syndrome. However, in older persons it is frequent the simultaneous presence of cognitive and motoric deficits. This new syndrome termed “motoric cognitive risk syndrome” has been recently proposed as a new risk factor for Alzheimer’s disease [6]. The detection of this condition, which requires information on subjective memory impairment or with “mild cognitive impairment” (MCI) and slow gait speed, helps, for instance, to better identify individuals at high risk of dementia, especially if older subjects [7].

Recently, the in vivo evaluation of amyloid deposition with Positron Emission Tomography (PET) has been introduced to anticipate the diagnosis of dementia, before symptoms become clinically evident [8–11]. Recent studies have found differences between subjects with age-associated cerebral amyloid deposition versus pathological deposition [12]. The current challenge is to address whether in older persons the amyloid deposition is associated with neuropsychological or motor impairment or is an expression of the aging process per se, and without influence on motoric and cognitive performance such as executive or memory function [13].

However, there is no current evidence in older persons with MCI of any correlation between amyloid PET and cognitive, motoric and behavioral functions. In this study, we therefore tested the hypothesis that amyloid cerebral deposition is correlated with the presence of reduced cognitive, behavior and physical performance in older persons with different combinations of impairments in cognitive, behavioral and physical function.

## Methods

We selected 48 elderly subjects (21 men and 27 women) with “*mild cognitive impairment (MCI)*” [14] evaluated at the Cognitive and Mobility Disorders Lab of the Department of Medicine, Geriatrics and Rehabilitation of the University-Hospital of Parma, and enrolled in the prospective observational T.R.I.P. Study (Traumatic Risk Identikit Parma Study) [15]. Briefly, patients at risk for falling were normally assessed by Comprehensive Geriatric Assessment (CGA) and those with MCI were enrolled in this study. In details, we consecutively selected subjects fulfilling the MCI “core” criteria as defined by the recommendations from the National Institute on Aging-Alzheimer’s Association (NIA-AA) workgroups on diagnostic guidelines for Alzheimer’s disease [14]. Inclusion criteria were a) MMSE score ≥ 24/30 in order to exclude demented persons [16] and b) concerns about cognitive modifications, expressed as subjective complaints by the subject or by impression by a close acquaintance or an expert clinician. Finally, participants with any memory complaint objectively confirmed or with the presence of a pathological Neuropsychiatric Inventory (NPI) questionnaire were considered [17]. Neuropsychiatric symptoms represent a common feature in addition to the full cognitive changes in dementia even in the mild cognitive impairment (MCI) setting [18]. Participants with established dementia, severe depression, or severe limitations in basic activities of daily living were excluded. Each patient had a recent brain computed tomography (CT) and/or magnetic resonance (MR) that excluded any secondary cause of cognitive impairment (*eg.* hydrocephalus, cerebral expansive lesions and stroke). Each patient was evaluated by a geriatrician with expertise in the administration of Comprehensive Geriatric Assessment and in particular of Mini Mental State Examination (MMSE), Clock Drawing Test (CDT), Activity Daily Living (ADL), Instrumental Activity of Daily Living (IADL), Neuropsychiatric inventory (NPI) questionnaire, Geriatric Depression Scale (GDS), Short Physical Performance Battery (SPPB) and handgrip strength [15]. All the selected subjects underwent amyloid-PET scan with 18F-flutemetamol to verify the presence of cerebral amyloid deposits (Aβ), in accordance with the indication provided by the Italian Ministry of Health.

In details, all patients were first evaluated by a trained geriatrician with a standard clinical evaluation [19] and, if necessary, also referred to a neuropsychologist with long-term experience in clinical and experimental neuropsychology of degenerative diseases [20]. The diagnosis of MCI due to AD or MCI not due to AD, was established using a standard evaluation protocol based on the new NIA-AA criteria [21]. Cognitive function was evaluated by Mini Mental State Examination and Clock Drawing Test. Mini Mental State Examination (MMSE) [16] consists of thirty items that refer to seven different cognitive areas: orientation in time and space, word recording, attention and calculation, re-evocation, language and executive function. The total score ranges between a minimum of 0 and a maximum of 30 points. The score is adjusted for age and the subject’s education. Clock Drawing Test (CDT) was used to assess executive cognitive dysfunction [22]. Many variants of the test are used in clinical practice: each variant proposes a different error detection with different scores that quantify them. In this study, it was chosen the clock test version in Camdex (watch dial to adjust = 1 point, all the numbers and hours correct position = 1 point, exact time = 1 point) (CDT equal to 0 means worst while 3 means the best performance) [23].

Depressive symptoms were assessed by the 15-item Geriatric Depression Scale (GDS-15) which detects changes in depressive symptoms after a major negative life event [24]. Physical performance was assessed by the Short Physical Performance Battery (SPPB) [25], while hand Grip strength was measured by manual dynamometer [26]. Neuropsychiatric symptoms were recorded by the neuropsychiatry inventory (NPI) scale [17]. All patients underwent a brain CT or MR scan in the previous 3 months. Cerebral atrophy and vascular cerebral changes on CT/MR scans was evaluated in accordance with the method reported by Ferguson et al. [27]. In details, cerebral atrophy included deep and superficial atrophy and vascular cerebral images included changes according to Fazekas’ scale. Missing data were integrated by checking original clinical sheets.

### 18F- Flutemetamol PET

Amyloid PET scans were performed using a whole-body hybrid system Discovery IQ (GE Healthcare) operating in three-dimensional detection mode. Head holder was used to restrict patient movement. Head movement was checked on a regular basis.

All cerebral emission scans began 90 min after a mean injection of 2 MBq/kg weight (150–250 MBq) of 18F-flutemetamol. For each subject, 10-min frames were acquired to ensure movement-free image acquisition. All PET sinograms were reconstructed with a 3-D iterative algorithm, with corrections for randomness, scatter, photon attenuation and decay, which produced images with an isotropic voxel of 2 × 2 × 2 mm and a spatial resolution of approximately 5-mm full-width at a half-maximum at the field of view center.

PET images were assessed visually by two trained, independent readers blinded each other with a previously described technique [28–30]. Images were reviewed in color, using a rainbow or Sokoloff color scale.

Regional quantification of 18F-Flutemetamol uptake was performed using a fully automated PET-only method as previously described by Thurfjell et al. [31] This technique is based on categorization of scans using a composite standardized uptake value ratio (SUVR) threshold derived from an autopsy cohort. The SUVRs in the cerebral cortex were generated automatically and normalized to the pons using the CortexID Suite software (https://www.gehealthcare.co.uk/-/media/13c81ada33df479ebb5e45f450f13c1b.pdf).

This software uses a threshold *z* score of 2.0 to indicate abnormally increased regional amyloid burden that corresponds to a composite SUVR of approximately 0.59 to 0.62 when normalized to the pons, providing a 99.4% concordance with visual assessment [31]. The study images were compared to the intrinsic software database control group (of > 100 amyloid negative flutemetamol healthy controls from GE Healthcare) as a whole to calculate the *z* scores compared to clinically negative amyloid scans.

Pathological amyloid-PET was defined when amyloid deposition involved at least one brain area with a *z-* score > 2.0. Normal tracer distribution at qualitative analysis with a threshold *z-*score less than 2.0 in all examined regions identified a negative amyloid-PET and considered as age-related amyloid cerebral deposition not considered as diseases.

The data were treated in agreement with Italian legislation on Data Protection. All participants provided written informed consent to participate. The study was approved by the Ethical Committee of the University Hospital of Parma (ID 17262 del 12/05/2017). It was conducted in compliance with the Good Clinical Practice.

### Statistical analysis

Data are reported as means and standard deviations or numbers and percentages. We defined subjects as *MCI due to AD* or *MCI not due to AD* in accordance to the presence or absence of pathological cerebral amyloid deposition evaluated by PET scan, respectively. In details, we detected amyloid deposition in the brain regions, schematically divided into frontal, temporal, parietal and occipital areas. Each of these four cerebral areas examined by amyloid PET was considered pathologically affected by amyloid deposition if the Z-score of the amyloid deposition in each area was greater than at least two times the normal reference population.

Then, we tested the difference between cognitive performance, behavioral and motoric impairment into the two groups (*MCI due to AD* versus *MCI not due to AD*) with the t-test of Student.

Furthermore, we performed a multivariable logistic regression analysis to study the relationship between cognitive performance, behavioral and motoric difference into the two groups (*MCI due to AD* versus *MCI not due to AD*).

Finally, we stratified the study sample in different categories in accordance to the contemporary presence of impaired clinical characteristics, for testing their relationship with the presence of pathological cerebral amyloid deposition. In details, we defined impaired CDT test if the subject had a score less than three, pathological NPI if the subject had a score more than 2, impaired physical performance if the SSPB score was less than 10, low grip strength if the score was less than 26 kg in men and 16 kg in women. Finally, we examined by age- years of school- and sex-adjusted logistic regression analysis the relationship of created pathological clinical categories and the presence or absence of pathological amyloid deposition, singularly. All *p*-values were considered significant for *p* < 0.05. The statistical processing was carried out using the Statistical Analysis System (SAS) 8.2 software.

## Results

The patients included in the study were 48, including 21 males (43.8%) and 27 females (56.2%). Mean age of the enrolled subjects was 74.6 ± 7.8 years (range 54–90 years) (Table 1).

**Table 1:** Characteristics of the study population (*n* = 48)

	All population		MCI due to AD	MCI not due to AD	t-value	*p*^a^
Age (years) (mean ± SD)	74.6 ± 7.8		76.0 ± 7.8	73.0 ± 7.5	−1.33	0.19
Sex (men) (n, %)	21	44.0	13 (52)	8 (35)	1.20	0.23
ADL (Katz’s scale) (mean ± SD)	5.3 ± 1.3		5.1 ± 1.4	5.4 ± 1.2	0.72	0.47
IADL (Lawton’s scale) (mean ± SD)	5.1 ± 2.6		4.6 ± 2.6	5.7 ± 2.6	1.46	0.15
MMSE (mean ± SD)	26.0 ± 2.0		24.7 ± 0.7	27.0 ± 1.0	1.30	0.20
Alterated Clock Drawing Test (CDT) (n, %)	29	61.0	18 (72)	11 (47)	**−2.72**	**0.03**
NPI (mean ± SD)	6.9 ± 5.9		9.4 ± 6.6	4.2 ± 3.6	**−3.40**	**0.006**
NPI (crude value), (n,%)						
2	16	33.33	5 (31)	11 (79)		
3	4	8.33	1 (25)	3 (75)		
4	5	10.42	3 (60)	2 40)		
6	6	12.50	2 (33)	4 (67)		
8	5	10.42	3 (60)	2 (40)		
12	5	10.42	5 (100)	0		
18	6	12.50	5 (83)	1 (17)		
24	1	2.08	1 (100)	0		
SPPB score (mean ± SD)	9.0 ± 2.6		9.0 ± 2.8	9.1 ± 2.5	0.0	0.99
Grip Strength (Kg) (mean ± SD)	25.6 ± 7.7		26.6 ± 8.3	24.7 ± 6.9	1.44	0.39
4-m Walking Speed (4-m WS) (m/sec) (mean ± SD)	0.87 ± 0.5		0.83 ± 0.4	0.88 ± 0.5	−0.61	0.54
Pathological Amyloid PET (n, %)	25	52.0	25 (52)	–		
Normal or Light alteration Amyloid PET^b^ (n, %)	23	48.0	–	23 (48)	–	
Cerebral Atrophy on CT^c^ (n, %)	26	54.0	11 (42)	14 (56)	1.48	0.15
Cerebral Vascular changes on CT^d^ (n, %)	31	64.5	16 (51)	9 (53)	0.09	0.93

^a^the difference between means was assessed with t-test of Student

^b^it means that the Z-score is less than 2 SD of the normal older population of reference

^c^described according to the reference [21]

^d^described according to the reference [21]

All of these subjects showed preserved cognitive function at MMSE (score corrected for age and education > 24), while the clock drawing test (CDT) was performed incorrectly in 29 of 48 subjects (61.0%).

The mean value of SPPB score was 9.0 ± 2.6, while the average muscle strength of the upper extremities measured by hand grip was 25.6 ± 7.7 Kg.

The average scores obtained in the ADL (Katz’s scale) was 5.3 ± 1.3, while in instrumental activities IADL (Lawton’s scale) equaled 5.1 ± 2.6. Finally, the mean NPI score was 6.9 ± 5.9 CT/MRI images showed cortical atrophic changes in 26 of the 48 examined subjects (54.0%), while cerebrovascular modifications were present in 31 subjects (64.5%).

Pathological burden of amyloid deposits was detected in 25 of 48 (52.0%) patients included in the study with a mean value of global z-score of 2.8 (subjects defined as MCI due to AD). Amyloid-PET was negative in 21 (44.0%) subjects and mild amyloid deposition (less than 1 z-score in each cerebral area) was present in 2 (0.5%) subjects (subjects defined as MCI not due to AD). We reported in Table 1 clinical, physical, cognitive and behavioral characteristics of the study sample after stratifying study sample in two these groups (MCI due to AD and MCI not due to AD). In details,

MCI due to AD showed significantly high percentage of subjects with not correct CDT (18, 72% versus 11, 47%; *p* = 0.03) and higher mean value of NPI (9.4 ± 6.6 versus 4.2 ± 3.6; *p* = 0.006) compared to MCI not due to AD. SPPB score, 4-m walking speed and grip strength were not significantly different between the two groups (Table 1).

Table 2 shows the multiple logistic regression analysis testing the difference between clinical, physical, cognitive and behavioral characteristics of the study sample after stratifying study sample in two groups (MCI due to AD and MCI not due to AD). Higher age and NPI were associated with an increased risk of having pathological deposition of cerebral amyloid deposits (O.R. = 1.18 [1.01–1.40], *p* = 0.03 and O.R. = 1.33 [1.05–1.68], *p* = 0.02, respectively). On the contrary, higher CDT score was associated with lower probability of having pathological amyloid PET deposition (O.R. = 0.48 [0.27–0.97], *p* = 0.04).

**Table 2:** Logistic regression analysis between MCI due to AD versus MCI not due to AD and clinical, cognitive and physical characteristics of the sample (*n* = 48)

	O.R.	95% CI	p
**NPI (Highest score is worse)**	**1.33**	**1.05–1.68**	**0.02**
**CDT (Highest score is better)**	**0.48**	**0.27–0.97**	**0.04**
ADL (Katz’s scale)	0.64	0.56–1.30	0.25
IADL (Lawton’s scale)	0.85	0.46–1.60	0.62
SPPB	1.27	0.77–2.09	0.34
Grip Strength	1.05	0.88–1.26	0.57
**Age**	**1.18**	**1.01–1.40**	**0.03**
Sex	1.85	0.08–44.3	0.70
Years of school	0.94	0.77–2.10	0.59
Cerebral Atrophy on CT/RM	0.32	0.05–1.92	0.21
Cerebral Vascular changes on CT/RM	0.85	0.46–1.60	0.62

Table 3 shows the percentage and association of subjects with more than one altered test, expression of cognitive, behavioral or physical performance evaluation with the probability of having MCI due to AD. Stratifying subjects in subclasses of clinical alterations, we found that subjects with impaired CDT and high NPI score showed more probability of having pathological amyloid deposition (O.R. = 3.45 [1.01–11.2], *p* = 0.04). Then, even subjects with both impaired CDT and low physical performance showed more probability of having pathological amyloid deposition (O.R. = 5.80 [1.04–32.2], *p* = 0.04). Similarly, subjects with alteration of CDT and high NPI score showed more probability of having pathological amyloid deposition (O.R. = 7.98 [1.38–46.0], *p* = 0.02). Finally, also subjects with alteration of CDT and high NPI and low physical performance showed more probability of having pathological amyloid deposition (O.R. = 5.80 [1.05–32.2], *p* = 0.04).

**Table 3:** Distribution of the study population stratified by cognitive and physical impairment and singular association with Pathological Amyloid PET

	N	%	O.R.	95%CI	p*
Altered CDT and NPI	23	48.0	**3.45**	**1.01–11.2**	**0.04**
Altered CDT and 4-m WS	6	12.5	0.46	0.07–3.07	0.42
Altered CDT and Low Grip Strength	4	8.3	3.03	0.28–33.4	0.36
Altered CDT and Low Physical Performance	13	27.1	**5.80**	**1.04–32.2**	**0.04**
Altered NPI and 4-m WS	4	8.3	0.29	0.02–4.16	0.36
Altered NPI and Low Grip Strength	5	10.4	4.12	0.38–44.5	0.24
Altered NPI and Low Physical Performance	18	37.5	**7.98**	**1.38–46.0**	**0.02**
Altered CDT and NPI and Low Physical Performance	13	27.1	**5.80**	**1.05–32.2**	**0.04**

*p is expression of singular logistic regression analysis between MCI due to AD versus MCI not due to AD for each reported categories

Singular analysis was also adjusted for age, sex and years of school

**Fig. 1:**
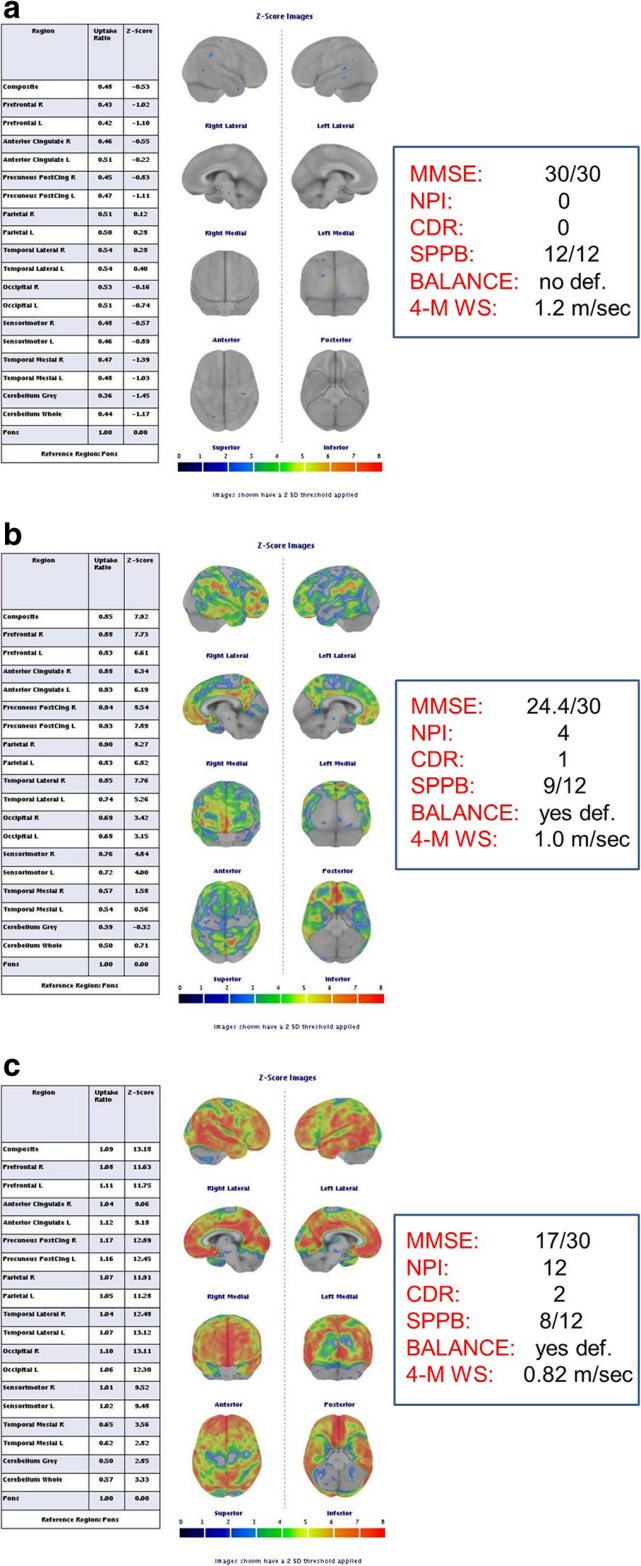
**a**, **b**, **c**. This figure shows an example of normal (**a**) and two amyloid pathological PET results (**b, c**). After considering only subjects with pathological PET examination, it emerges from the mean of the uptake values of 18F-Flutemetamol a widespread and pathological deposition of cerebral amyloid. In the first patient (**b**) a lower involvement of sensory-motor regions of the right and left (average values of z-score of 3.25 ± 2.44 and 3.01 ± 2.42, respectively), occipital left and right (average values of z-score of 3.83 ± 3.75 and 3.15 ± 3.08, respectively) and right and left medial temporal region which remains the cortical area less involved by amyloid pathology (mean values of z-score of 0.83 ± 1.59 and 0.47 ± 1.60, respectively). In the second patient (**c**), the amyloid deposition is more abundant probably expression of advanced disease. There is a higher involvement of sensory-motor regions of the right and left (average values of z-score of 9.52 ± 1.01 and 9.45 ± 1.02, respectively), and occipital regions of the right and left (average values of z-score of 13.11 ± 1.10 and 12.30 ± 1.06, respectively) and right and left medial temporal region which remain the cortical area less involved by amyloid pathology (mean values of z-score of 3.56 ± 0.65 and 2.82 ± 0.62, respectively)

In Fig. 1(a,b,c) we show an example of negative (Fig. 1a) and two positive amyloid-PET results (Fig. 1b and c). By taking into account only subjects with pathological PET examination, we found an higher mean of the uptake values of 18F-Flutemetamol indicating the widespread and pathological deposition of cerebral amyloid. In the first patient (1b) a lower involvement of sensory-motor regions of the right and left (average values of z-score of 3.25 ± 2.44 and 3.01 ± 2.42, respectively), occipital left and right (average values of z-score of 3.83 ± 3.75 and 3.15 ± 3.08, respectively) and right and left medial temporal region which remains the cortical area less involved by amyloid pathology (mean values of z-score of 0.83 ± 1.59 and 0.47 ± 1.60, respectively). In the second patient (1c), the amyloid deposition is more abundant and is a probable expression of advanced disease. There is a higher involvement of sensory-motor regions of the right and left (average values of z-score of 9.52 ± 1.01 and 9.45 ± 1.02, respectively), and occipital regions of the right and left (average values of z-score of 13.11 ± 1.10 and 12.30 ± 1.06, respectively) and right and left medial temporal region which remains the cortical area less involved by amyloid pathology (mean values of z-score of 3.56 ± 0.65 and 2.82 ± 0.62, respectively).

## Discussion

This study shows that patients with MCI due to AD and cerebral amyloid deposition have a high amount of amyloid plaques in brain cortical structures, not only in the temporal lobes. This feature could explain some of MCI clinical manifestations that may be attributable not only to the disturbance of memory. MCI in older persons could affect different cognitive domains and the global brain involvement demonstrated by amyloid-PET is associated with the impairment of several higher brain activities, and in particular executive functions, behavior and probably physical performance. In other words, our findings show also that when amyloid deposition is pathologically present, which is expressed by high z-score at the PET scans, brain lobes are globally involved and cognitive performance captured by MMSE score could remained almost normal. Executive function, mood behavioral symptoms and physical performance could be compromised.

These findings support recent data showing that pathological amyloid deposition could be present at least several years before the appearance of clinical manifestation of the AD [32]. Amyloid-PET could be used together with clinical, motor and objective neuropsychological tests for early diagnosis of MCI due to AD [33], even if MMSE expression of global cognitive function is normal [34]. Our results are in accordance with previous studies showing that executive functions, evaluated by CDT [35] or Montreal Cognitive Assessment (MoCA) tests [36], plus physical performance impairment could identify persons at risk of developing AD [7].

Another interesting aspect emerging from our results is that CDT correlates with amyloid pathology within almost normal MMSE values and this could be explained by the fact that CDT requires activation of various neuro-psychological functions, such as auditory perception, auditory memory, abstraction ability, visual memory, visual perception, visual-spatial functions, planning capacity, visual motor and executive functions [34–36]. This association could be related to the global amyloid deposition in the central nervous system and supports the evidence that the cerebral amyloid angiopathy (CCA) could be the result of a deficit of the protein clearance pathways [37]. Recently, Morris et al. [38] proposed that an impairment of the cerebral vascular basement membranes by which fluid passes into and out of the brain explains the accumulation of the amyloid in the central nervous system with an imbalance between production and its clearance.

Our data also suggest that if amyloid deposit if clinically present, the amount of deposition should be relevant for being detected and the process could influence different brain areas and not only the medial temporal lobe where important memory related circuits are located. This aspect is in line with other studies [39]. In fact, the hierarchical amyloid deposition in the brain had already been suggested in the past by Thal and Braak [40] from pathological studies, suggesting that the onset of aggregates of β-amyloid initially affected areas of the neocortex as the frontal, parietal and temporal area and only in more advanced stages the hippocampus would be affected by the amyloid pathology. It could be speculated that in the early phase of the disease the deposition of amyloid should involve globally the cortex, and this process could require several years. Recently, a longitudinal study realized in cognitively normal older persons, showed that higher amyloid beta burden was associated with increasing anxious-depressive symptoms over time [41], and these results are consistent with our data showing that amyloid brain deposition produces a behavior modification, particularly evident in the atypical forms of dementia.

Then, probably only when the amount of amyloid is high enough, Tau deposits are produced from amyloid in areas such as the medial temporal lobe, with clinical picture of loss of long-term memory. This hypothesis is in accordance with recent results reported by Donohue et al. [42], showing that there is a time window of at least 5 years between the initial deposition of amyloid and a clear reduction of cognitive performance detected by MMSE. More recently, even findings reported by Sepulcre J et al. [43] confirm this hierarchical organization of Tau and Amyloid deposits in the cerebral cortex. In particular, these authors suggest that several years before AD dementia manifestations, abnormal accumulations of tau and Aβ insoluble proteins are visible in the temporal lobe and association cortex. Tau and Aβ deposits show some degree of spatial specificity as well as some overlapping in convergent zones [44].

To date, research in the field of AD “causal” therapy is increasingly directed to identify subjects with AD in the pre-clinical stage, when amyloid deposits in the cortex are scarce and cognitive function not compromised. Hence, the possibility of having AD biomarkers in pre-clinical phase appears increasingly important for better understanding the progression from MCI to dementia, and identifying cluster of markers that intercept patients’ candidate for prescription of future drugs will be fundamental. Among these biomarkers of AD, the amyloid PET, could be useful for really identifying *pre-mortem* patients with high probability to be affected by AD. Our results support this hypothesis especially when the amyloid deposition is plentiful, as in our study, and higher than 2 times amount in age-matched older persons, it should not be considered as the “normal” effect of aging. Conversely, the presence of high amyloid burden with global diffusion in the cerebral cortex is associated with an increased risk of developing AD over time [45, 46].

Recent data support a dose-response relationship between amyloid deposition and cognitive performance [47]. The authors found that the magnitude of amyloid burden at baseline was associated with the rate of cognitive decline over 4-year follow-up period, suggesting a potential link between these two phenomena. Our findings may have important implications also for projecting clinical outcomes on amyloid-PET scan basis, as well as for understanding the effect of amyloid in preclinical AD.

The clinical significance of these results in the routine evaluation of AD patients is confirmed by a recent PET study that showed that, in almost a quarter of selected patients, [18F] flutemetamol PET changed the clinical diagnosis and altered the patient management plan. The authors concluded that amyloid-PET might have added benefit over the standardized diagnostic work-up in early-onset dementia patients with uncertain clinical diagnosis, providing evidence for the recommendations put forward in the appropriate use criteria for amyloid PET in clinical practice [48–50].

The main limitation of our study is the small number of patients that should be considered to draw definitive conclusions. Furthermore, selection of older persons with MCI could produce a significant “ceiling effect” reducing diagnostic accuracy of cognitive impairment. Thus, persons were referred to our Lab because at high of falls and this could represent a cohort bias of selection, our results could not be considered as representative of older persons.

Despite these limitations, this study supports the idea that even MCI due to AD is a multi-domain disease that affects the cognitive sphere, neuropsychiatric and physical performance aspects of the persons affected with loss of autonomy initially in performing instrumental activities of daily living. All these aspects could be of importance during the initial evaluation of the patients. Through this study, we reinforce the hypothesis of a hierarchical deposition of amyloid aggregates, and the role of major cortical involvement of amyloid pathology in determining greater cognitive and functional impairment [7].

## Data Availability

The datasets used and/or analyzed during the current study are available from the corresponding author on request.

## Abbreviations

MCI: Mild cognitive impairment
AD: Alzheimer’s disease
PET: Positron Emission Tomography
CGA: Comprehensive geriatric assessment
MMSE: Mini Mental State Examination
CDT: Clock Drawing Test
ADL: Activity Daily Living
IADL: Instrumental Activity of Daily Living
NPI: Neuropsychiatric inventory
GDS: Geriatric Depression Scale
SPPB: Short Physical Performance Battery
MoCA: Montreal Cognitive Assessment
